# Effect of lean status on the mortality of patients with non-alcoholic fatty liver disease (NAFLD): A systematic review and meta-analysis

**DOI:** 10.12669/pjms.41.6.12044

**Published:** 2025-06

**Authors:** Ping Miao, Zhongyi Shao, Ji Jiang

**Affiliations:** 1 Ping Miao Traditional Chinese Medicine Diagnosis and Treatment Centre, Affiliated People’s Hospital of Ningbo University, Ningbo, Zhejiang Province 315040, P.R. China; 2 Zhongyi Shao Department of Pharmacy, Affiliated People’s Hospital of Ningbo University, Ningbo, Zhejiang Province 315040, P.R. China; 3 Ji Jiang Department of Emergency, Affiliated People’s Hospital of Ningbo University, Ningbo, Zhejiang Province 315040, P.R. China

**Keywords:** All-cause mortality, Systematic review, Body mass index, Lean, Meta-analysis, NAFLD, Non-alcoholic fatty liver disease, Overweight, Obese

## Abstract

**Objective:**

To investigate the impact of lean status as per body mass index (BMI) on mortality in cases of non-alcoholic fatty liver disease (NAFLD).

**Methods:**

A systematic search was conducted in PubMed, Embase, and Scopus to identify relevant studies published from the inception of each database up to July 31, 2024. Observational studies that reported data on the mortality outcomes of NAFLD patients with different BMI, and provided adjusted estimates were included. The Newcastle-Ottawa Scale (NOS) was used for quality assessment. Pooled effect sizes were reported as hazard ratio (HR) with 95% confidence intervals (CI).

**Results:**

Fourteen studies were included, of which majority were retrospective cohort studies (n=10). Objective assessment method for NAFLD i.e., imaging and/or biopsy was used in 10 studies. Compared to NAFLD patients with high BMI, lean patients had higher risk of all-cause mortality (HR 1.34, 95% CI: 1.23, 1.47), with no publication bias for any of the outcomes. Lean status correlated with elevated risk of liver disease-related mortality (HR 2.14, 95% CI: 1.18, 3.87) but had similar risk of cardiovascular (HR 0.92, 95% CI: 0.65, 1.30) and cancer-related mortality (HR 1.20, 95% CI: 0.90, 1.60).

**Conclusion:**

Lean status of NAFLD patients correlates with higher risk of all-cause and liver disease-related mortality compared to patients with high BMI. There is a need for tailored interventions and further research to understand specific mortality risks in this subgroup of patients.

## INTRODUCTION

Incidences of non-alcoholic fatty liver disease (NAFLD) are on the rise, from nearly 25% in 1990-2006 to 38.0% in 2016-2019.^1^ NAFLD is more frequent in males, compared to females,^2^ and correlates with adverse metabolic health outcomes such as obesity, Type-2 diabetes, hypertension, and dyslipidemia.^3^,^4^ The studies also indicate its potential for progression to severe conditions like liver fibrosis, cirrhosis, hepatocellular carcinoma (HCC), and, ultimately, mortality.^5^-^7^

While obesity is a prominent contributor to NAFLD, around two-fifth (40%) of NAFLD patients are nonobese, and 20% fall under the lean category, with body mass index (BMI) below 23 kg/m^2^ in Asian populations and below 25 kg/m^2^ in other ethnicities.^8^,^9^ Studies indicate that lean patients with NAFLD often exhibit more favourable metabolic profiles, and fewer incidences of fibrosis compared to patients with higher BMIs.^10^–^13^ However, the link between lean status and mortality, and liver-related outcomes in NAFLD patients is still controversial.^14^–^16^

It is important to note that in the recent years, the nomenclature surrounding NAFLD has evolved to better reflect the underlying pathophysiology and to reduce diagnostic ambiguity. Initially redefined as metabolic dysfunction-associated fatty liver disease (MAFLD), the terminology has more recently been updated to metabolic dysfunction-associated steatotic liver disease (MASLD), with the term metabolic dysfunction-associated steatohepatitis (MASH) now used to describe its inflammatory subtype. These changes aim to emphasize the metabolic underpinnings of the disease and provide a more inclusive and pathophysiologically relevant framework. However, given that the studies included in this review were conducted using the previous nomenclature, we have retained the term “NAFLD” throughout this manuscript to maintain consistency with the original study definitions and ensure clarity in interpretation.

Three systematic reviews conducted by Ha et al. (2023), Wongtrakul et al. and Huang et al.)^14^-^16^ addressed the issue of mortality risks in lean NAFLD patients. While each review presents unique findings, they all have common methodological limitations that potentially affect the accuracy of reported associations. Ha et al. review, which encompassed 10 cohort studies showed comparable risks for all-cause mortality and cardiovascular mortality in NAFLD patients with different BMI. However, lean status in NAFLD patients correlated with notably higher risk of liver-related mortality.^14^ Notably, the review included studies with unadjusted estimates, possibly introducing confounding factors that could influence the reported associations. Moreover, the inclusion of a study comparing mortality risks in lean subjects with and without NAFLD raises questions about the appropriateness of the comparator.^17^

Similarly, the review of Wongtrakul et al. involving 14 studies, pooled unadjusted estimates and included studies with inappropriate comparators and conference abstracts.^15^ Wongtrakul et al. reported higher all-cause mortality in lean *versus* high-BMI NAFLD patients. However, both groups had comparable risks of cardiovascular and malignancy-related mortality.^15^ The third review by Huang et al which incorporated seven studies, indicated that lean patients with NAFLD had a 1.4 times higher risk of all-cause mortality.^16^ This review mirrored the limitations observed in the previous reviews, raising concerns about the consistency and reliability of the reported associations. Notably, while all three reviews were conducted around the same time, there was a discrepancy in the number of included studies, which raises doubts about the adopted methodology and overall interpretation of findings.

In the light of these considerations, this meta-analysis aimed to meticulously identify all relevant studies that report confounder adjusted estimates and undertake a comprehensive examination of the effect of BMI on the mortality rates in NAFLD patients.

## METHODS

### Eligibility criteria (PECOS Framework):

*Participants(P):* Adults diagnosed with non-alcoholic fatty liver disease (NAFLD), regardless of geographic location or clinical setting.

*Exposure(E):* Lean status, defined as a body mass index (BMI) below the threshold specified by individual studies (typically <25 kg/m² for non-Asian populations and <23 kg/m² for Asian populations).

*Comparator(C):* Non-lean individuals with NAFLD, including those categorized as overweight or obese based on BMI thresholds defined by each study.

*Outcomes(O):* The primary outcome was all-cause mortality. Secondary outcomes included liver disease-related mortality, cardiovascular mortality, and cancer-related mortality. Only studies reporting adjusted risk estimates (e.g., HRs or ORs) accounting for potential confounders were included.

*Study design (S):* Observational studies, including prospective or retrospective cohort studies and case-control studies, published up to July 31, 2024. Only peer-reviewed studies adhering to COPE (Committee on Publication Ethics) guidelines were considered.

### Exclusion criteria:

- Studies without clear definitions of lean and non-lean categories.
- Studies that did not stratify mortality risk based on lean status.
- Studies focusing on liver diseases unrelated to NAFLD.
- Studies using comparators other than non-lean/overweight/obese individuals with NAFLD.
- Case reports, review articles, conference abstracts, or studies published in journals lacking a peer-review process or non-compliant with COPE guidelines

### Information sources:

PubMed, Embase, and Scopus databases were explored. Eligible studies for inclusion were those published up to July 31, 2024. Additionally, a review of the reference lists of included studies was conducted as a supplementary step to identify any potentially overlooked studies.

### Search strategy:

The search strategy consisted to the key words: (“nonalcoholic fatty liver disease” OR “NAFLD” OR “fatty liver disease” OR “metabolic dysfunction-associated fatty liver disease” OR “MAFLD”) AND (“mortality risk” OR “mortality” OR “death” OR “survival” OR “fatal outcome” OR “all-cause mortality” OR “cardiovascular mortality” OR “liver-related mortality” OR “malignancy-related mortality”) AND (“underweight” OR “low weight” OR “thin” OR “normal weight” OR “lean” OR “Lean BMI”) AND (“overweight” OR “obese” OR “excess weight” OR “high BMI” OR “body mass index”). The detailed search strategy for each of the databases is mentioned in Supplementary file-1.

### Selection process:

After compiling the initial set of studies, identified by the literature search, duplicates were removed. Following this step, two authors (ZS and JJ) independently conducted a review of the remaining studies. During the initial screening phase, studies that showed relevance based on title and abstract underwent further assessment, which included reading the full texts. Any discrepancies in decisions regarding study inclusion were resolved by consensus. These discussions were moderated by the senior author (PM)

### Data collection process:

A pre-developed structured form that was pilot-tested, and refined, was used by two authors (ZS and JJ) to independently extract relevant data. Any disagreements regarding extracted data were resolved through consensus, with discussions facilitated by the senior author (PM).

### Risk of bias assessment:

Quality assessment was done using the Newcastle-Ottawa Scale (NOS) by the two authors (ZS and JJ).^18^ The Newcastle–Ottawa Scale (NOS) is a widely accepted tool for evaluating the methodological robustness of non-randomized studies. It assesses studies across three broad domains:

### Selection:

which evaluates how well the study groups are defined and representative;

### Comparability:

which examines whether the studies adequately control for potential confounders; and

### Outcome

(for cohort studies) or Exposure (for case-control studies): which assesses the methods used to ascertain outcomes and the adequacy of follow-up. Each domain is scored based on specific criteria, with a maximum total score of nine. Studies that score higher on the NOS are considered to have better methodological quality and lower risk of bias. Any disagreements regarding quality assessments were resolved through consensus, with discussions facilitated by the senior author (PM). Adherence to the PRISMA guidelines was maintained,^19^ and the protocol was prospectively registered at PROSPERO (registration number: CRD42024512065).

SUPPLEMENTARY FILE-1

***Search Strategy for Systematic Review and Meta-analysis:***

This document outlines the detailed electronic search strategies used for the systematic review and meta-analysis titled ‘Effect of lean status on the mortality of patients with non-alcoholic fatty liver disease (NAFLD)’. The search was conducted in PubMed, Embase, and Scopus databases from their inception until July 31, 2024. The following keywords and Boolean operators were used to identify relevant studies.

***PubMed Search Strategy:***

(“nonalcoholic fatty liver disease” [Title/Abstract] OR “NAFLD”[Title/Abstract] OR “fatty liver disease”[Title/Abstract] OR “metabolic dysfunction-associated fatty liver disease”[Title/Abstract] OR “MAFLD”[Title/Abstract]) AND (“mortality risk”[Title/Abstract] OR “mortality”[Title/Abstract] OR “death”[Title/Abstract] OR “survival”[Title/Abstract] OR “fatal outcome”[Title/Abstract] OR “all-cause mortality”[Title/Abstract] OR “cardiovascular mortality”[Title/Abstract] OR “liver-related mortality”[Title/Abstract] OR “malignancyrelated mortality”[Title/Abstract]) AND (“underweight”[Title/Abstract] OR “low weight”[Title/Abstract] OR “thin”[Title/Abstract] OR “normal weight”[Title/Abstract] OR “lean”[Title/Abstract] OR “Lean BMI”[Title/Abstract]) AND (“overweight”[Title/Abstract] OR “obese”[Title/Abstract] OR “excess weight”[Title/Abstract] OR “high BMI”[Title/Abstract] OR “body mass index”[Title/Abstract])

***Embase search strategy:***

(“nonalcoholic fatty liver disease”:ab,ti OR “NAFLD”:ab,ti OR “fatty liver disease”:ab,ti OR “metabolic dysfunction-associated fatty liver disease”:ab,ti OR “MAFLD”:ab,ti) AND (“mortality risk”:ab,ti OR “mortality”:ab,ti OR “death”:ab,ti OR “survival”:ab,ti OR “fatal outcome”:ab,ti OR “all-cause mortality”: ab,ti OR “cardiovascular mortality”:ab,ti OR “liver-related mortality”:ab,ti OR “malignancy-related mortality”:ab,ti) AND (“underweight”:ab,ti OR “low weight”:ab,ti OR “thin”:ab,ti OR “normal weight”:ab,ti OR “lean”:ab,ti OR “Lean BMI”:ab,ti) AND (“overweight”:ab,ti OR “obese”:ab,ti OR “excess weight”:ab,ti OR “high BMI”:ab,ti OR “body mass index”:ab,ti)

***Scopus search strategy:***

TITLE-ABS-KEY(“nonalcoholic fatty liver disease” OR “NAFLD” OR “fatty liver disease” OR “metabolic dysfunction-associated fatty liver disease” OR “MAFLD”) AND TITLE-ABS-KEY(“mortality risk” OR “mortality” OR “death” OR “survival” OR “fatal outcome” OR “all-cause mortality” OR “cardiovascular mortality” OR “liver-related mortality” OR “malignancy-related mortality”) AND TITLE-ABS-KEY(“underweight” OR “low weight” OR “thin” OR “normal weight” OR “lean” OR “Lean BMI”) AND TITLE-ABS-KEY(“overweight” OR “obese” OR “excess weight” OR “high BMI” OR “body mass index”).

### Statistical analysis:

STATA version 15.0. was used. The pooled effect size was presented as hazard ratio (HR) along with 95% confidence intervals (CI). A random-effects model was employed when Cochrane I^2^ exceeded 40%, indicating substantial heterogeneity.^20^ Subgroup analysis for the primary outcome i.e., all-cause mortality was conducted based on study design, diagnostic method for NAFLD, sample size, duration of follow up and study setting. Publication bias was determined based on Egger’s test.^21^ P <0.05 indicated statistical significance.

## RESULTS

**Fig.1:**
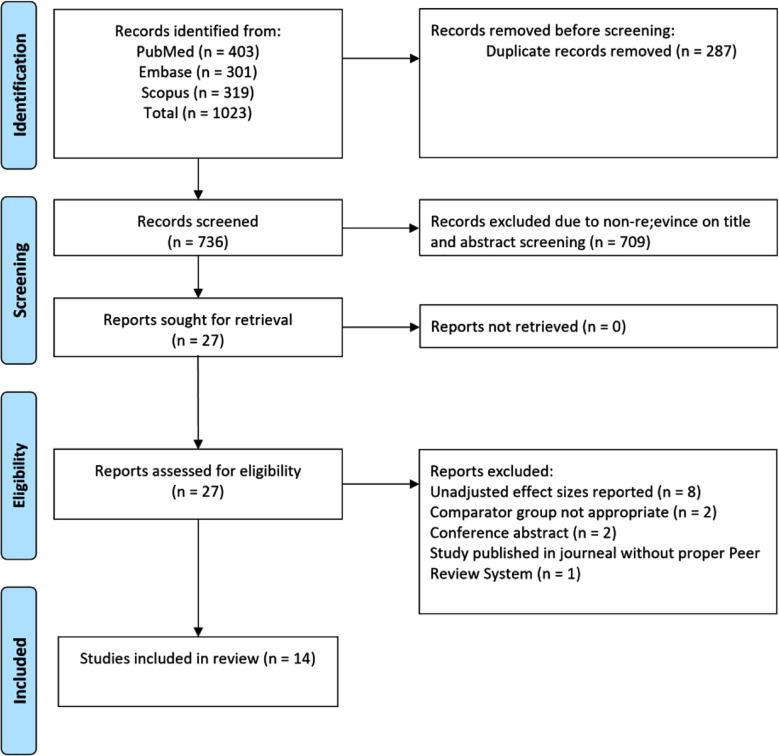
Selection of studies.

Electronic search of the databases identified 1023 studies. Subsequently, 287 duplicate studies were removed (Fig.1), and titles and abstracts of the remaining 736 studies were screened. Following screening, 709 studies were excluded, and full texts of the remaining 27 studies were read. The final analysis incorporated a total of 14 eligible studies (Fig.1).^22^-^35^

The included studies were mostly retrospective cohort in design (n=10) Table-I. The remaining four studies has a prospective design. Most studies were from the United States of America (n=5) followed by two studies from Republic of Korea. One study each was conducted in France, China, Thailand, Austria, Japan and Sweden. Objective assessment method for NAFLD i.e., imaging and/or biopsy was used in 10 studies. In the remaining four studies, either a composite score or index based on metabolic markers or ICD-9-CM code- based diagnosis was made Table-I. Most included studies consistently categorized lean patients as having a BMI of less than 23 kg/m^2^ for Asian population and BMI less than 25 kg/m^2^ for patients from high-income settings. The duration of follow up ranged from 3.5 years to 20 years. In most studies, the age of the patients was between 40 to 55 years and males constituted around 45 to 75% of the sample. Total sample size of the included studies was 32,03,848 patients Table-I. Mean NOS score was 7.50, with seven studies obtaining a score of eight and seven - a score of seven.

**Table-I:** Summary of the included studies.

Author	Study design; location	Age and sex of participants	Diagnosis of NAFLD	Definition of lean	Sample size and follow up duration	NOS score
Chung (2023)^22^	RC; Republic of Korea	Mean age 50 years Male (76%)	Scoring with presence of metabolic abnormalities	BMI<23 kg/m2	30,15,286 Median 9.3 yrs	8
Nguyen (2023)^23^	RC; United States of America	Mean age 50 years; male (45%)	Liver biopsy	BMI <23 kg/m² for Asian ethnicity and <25kg/m² for other ethnicities	9061 Mean 41 months	8
Dao (2023)^24^	RC; United States of America	Median age of 49 years; Male (55%)	Liver USG along with metabolic risk abnormalities	BMI<30 kg/m2	2619 Follow up of around 20 years	8
Wijarnpreecha (2023)^25^	RC; United States of America	Median age of 51 years; Male (45%)	Imaging or biopsy	BMI<23 kg/m2 for Asians BMI<25 kg/m2 for other ethnicity	18,594 Median follow up of 49.3 months	7
Nabi (2023)^26^	PC; France	Mean age 47 years; male (46%)	Score/index based	BMI <23 kg/m² for Asian ethnicity and <25kg/m² for other ethnicities	25,753 Median 3.75 years	7
Younes (2022)^27^	PC; Multicentric	Mean age 48 years; male (65%)	Biopsy	BMI<25 kg/m2	1339 Median 7.7 years	7
Lan (2022)^28^	PC; China	Mean age 53 years Male (75%)	Abdominal USG	BMI<23 kg/m2	23197 Mean of 13.1 years	8
Ahmed (2022)^29^	RC; United States of America	Mean age of 51 years; male (44%)	Biopsy/imaging or cirrhosis with associated metabolic comorbidities	BMI<23 kg/m2 for Asians and Pacific Islanders BMI<25 kg/m2 for others	3645 Median follow up of 6.4 years	7
Charatcharoenwitthaya (2022)^30^	RC; Thailand	Mean age 49 years; male (31%)	Score/index based	BMI <23 kg/m2	7083 Mean 8.5 years	7
Feldman (2021)^31^	RC; Austria	Mean age 49 years; male (72%)	Biopsy	BMI≤25 kg/m2	299 Mean 8.4 years	8
Zou (2020)^32^	RC; United States of America	Majority aged over 50 years (63%) Male (53%)	Score/index based	BMI<27.5 kg/m2 for Asians BMI<30 kg/m2 for non-Asians	4711 Follow up of 15 years	8
Hirose (2020)^33^	RC; Japan	Mean age 43 years; male (63%)	Biopsy	BMI<25 kg/m2	223 Mean 19.5 years	7
Chang (2019)^34^	PC; Republic of Korea	Mean age 41 years; male (77%)	Ultrasound	BMI<25 kg/m2	91,392 Median 5.2 years	7
Hagström (2017)^35^	RC; Sweden	Mean age 49 years Male (62%)	Liver biopsy	BMI<25 kg/m2	646 Mean of 19.9 years	8

RC- retrospective cohort; PC-prospective cohort; BMI- body mass index; T2DM- type 2 diabetes mellitus; NAFLD- non-alcoholic fatty liver disease

### Risk of all-cause mortality:

**Fig.2:**
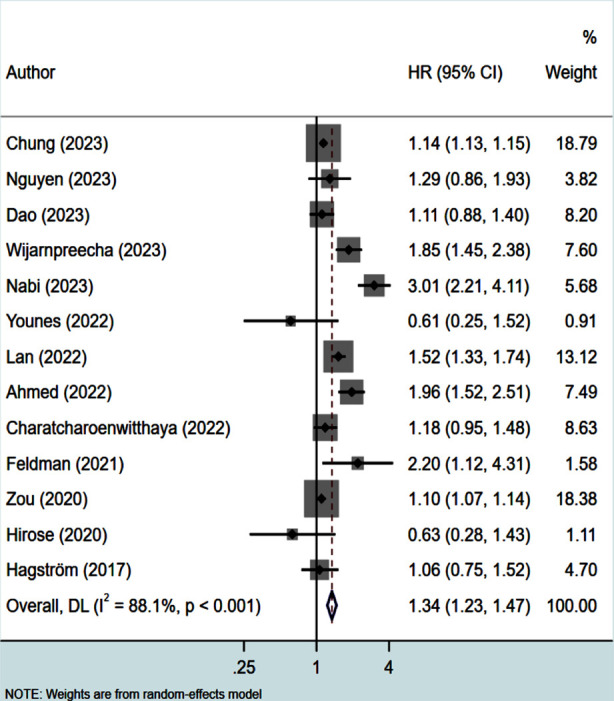
Risk of all-cause mortality (ACM) in lean subjects with NAFLD, compared to non-lean subjects with NAFLD.

Lean NAFLD patients had higher risk of all-cause mortality (HR 1.34, 95% CI: 1.23, 1.47; N=13, I^2^=88.1%) (Fig.2) compared to non-lean individuals, with no evidence of publication bias. (Egger’s p value 0.20). The subgroup analysis showed increased risk of all-cause mortality (ACM) when studies with a retrospective design, larger sample size (≥2000), longer follow up duration (≥10 years) and done in non-Asian settings were pooled together (Table-II). The association of lean status with higher risk of mortality was noted irrespective of the method used for diagnosis of NAFLD (Table-II).

**Table-II:** Subgroup analysis.

Subgroups	All-cause mortality
	HR with 95% CI (Number of studies; I^2^)
***Study design***	
Retrospective cohort	1.28 (1.19, 1.38) (10; 85.6%) *
Prospective cohort	1.60 (0.86, 2.99) (3; 90.3%)
***Diagnosis of NAFLD***	
Imaging or biopsy	1.38 (1.13, 1.69) (9; 70.5%) *
Other methods†	1.29 (1.17, 1.41) (4; 94.1%) *
***Sample size***	
≥2000	1.39 (1.28, 1.52) (9; 90.5%) *
<2000	1.03 (0.61, 1.72) (4; 60.0%)
***Duration of follow up***	
≥10 years	1.56 (1.27, 1.92) (5; 82.7%) *
<10 years	1.16 (0.95, 1.42) (8; 92.0%)
***Study setting***	
Asian	1.22 (1.00, 1.49) (4; 84.7%)
Non-Asian (USA, France, Australia, Austria, Sweden)	1.47 (1.20, 1.81) (9; 91.4%) *

* indicates statistical significance at p<0.05; †included scoring or index based on metabolic markers/ICD-code based

### Risk of cardiovascular, liver and cancer-related mortality:

**Fig.3:**
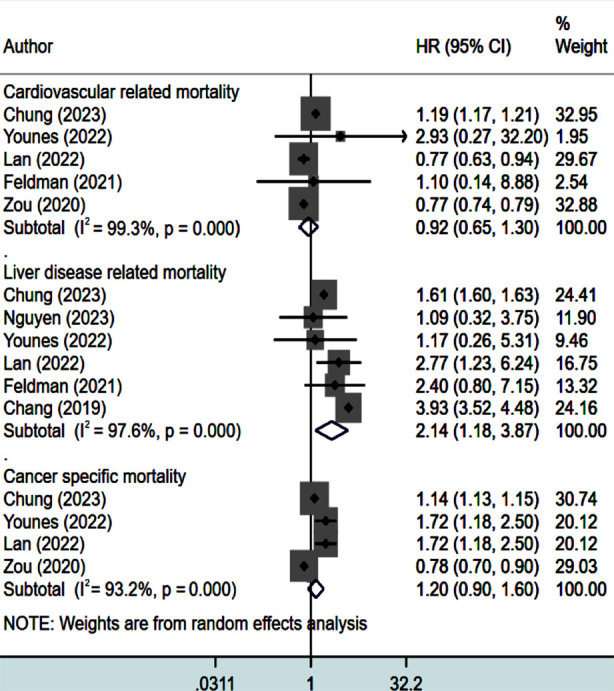
Risk of cardiovascular, liver and cancer-related mortality in lean subjects with NAFLD, compared to non-lean subjects with NAFLD.

Lean status of NAFLD patients was linked to significantly higher risk of liver disease-related mortality (HR 2.14, 95% CI: 1.18, 3.87; N=6, I^2^=97.6%) (Fig.3). However, risk of cardiovascular (HR 0.92, 95% CI: 0.65, 1.30; N=5, I^2^=99.3%) and cancer-related mortality (HR 1.20, 95% CI: 0.90, 1.60; N=4, I^2^=93.2%) was comparable in both groups (Fig.3). There was no evidence of publication bias for these outcomes, both on Egger’s test (p=0.62 for cardiovascular mortality; 0.37 for liver related mortality and 0.88 for cancer related mortality).

## DISCUSSION

Our analysis showed a significant link between lean status and mortality risk in NAFLD patients. Specifically, lean patients with NAFLD were at higher risk of all-cause mortality. Lean status of NAFLD patients was also associated with a markedly higher risk of liver disease-related mortality. In contrast, risk of cardiovascular and cancer-related mortality was comparable in both groups.

Our findings are largely in line with the previous reviews on this issue.^14^–^16^ While the exact reason for the increased mortality risk in lean NAFLD patients remains unclear, it may be due to elevated risks of significant liver-related adverse outcomes. Studies in Sweden and China indicate that lean NAFLD patients have higher incidence of cirrhotic liver changes and decompensated liver disease, leading to increased liver-related mortality.^28^,^35^ This difference may be genetic, and more specifically, linked to the patatin-like phospholipase domain-containing 3 (PNPLA3) genotype. Lean status in NAFLD is associated with higher frequency of PNPLA3 GG genotype, known to modify NAFLD progression.^36^–^38^ This underscores the need for deeper understanding of genetic factors and molecular mechanisms that lead to the heightened mortality risk in this population.

Studies have also examined the risk of mortality in lean subjects with MAFLD, compared to non-lean counterparts. A recent study that used Korean National Health Insurance Service database found that MAFLD-lean groups had a higher risk of all-cause and disease-specific mortality than did the MAFLD-overweight/obese group.^22^ Similarly, a systematic review by Sato-Espinoza et al documented lean MAFLD patients exhibited a worse prognosis compared to the obese or overweight counterpart.^39^

Previous studies have reported good cardiometabolic profiles of lean NAFLD patients.^12^,^14^ A meta-analysis that included 85 studies showed that lean NAFLD patients have fewer metabolic dysfunctions compared to overweight or obese patients.^40^ Our analysis found that while lean status was associated with fewer metabolic comorbidities in NAFLD patients, their risk of cardiovascular-related mortality was statistically similar to that of NAFLD patients with higher BMI. Long-term cohort studies and general population data support these findings, suggesting that despite having a healthier baseline status lean NAFLD patients have similar risks of cardiovascular-related morbidity and mortality as non-lean patients.^41^,^42^

We may also speculate that one of the mechanisms of the observed effect is due to the role of visceral adipose tissue, known for its metabolic activity and heightened inflammation potential,^43^ in the progression of NAFLD.^44^,^45^ It is noted that lean NAFLD patients can have the same visceral adiposity as non-lean or obese patients.^46^ Therefore, using, indices that reflect visceral fat content, rather than BMI, may provide a more robust stratification of NAFLD patients, and may be a better predictor of clinical prognosis.

Previous studies have showed variability in the intestinal microbiota between NAFLD patients with different BMI.^47^,^48^ Although not conclusively established, it is plausible that this difference may have some connection to the difference in mortality risks in these two groups of patients. For example, variability in the levels of Ruminococcaceae and Veillonellaceae species that inversely correlate with the degree of liver fibrosis, have been shown to be more pronounced in non-lean NAFLD patients.^49^ Another study highlighted a significant deficit of Ruminococcaceae in lean patients with non-alcoholic steatohepatitis.^48^ This deficiency might contribute to the elevated liver-related mortality observed in lean NAFLD patients.

In summary, the relationship between BMI and NAFLD-related mortality is underpinned by complex and multifactorial pathophysiological mechanisms. In lean individuals with NAFLD, mortality risk may be driven by a distinct set of mechanisms. These include genetic predispositions, sarcopenia, altered gut microbiota, and subtle but significant metabolic dysfunction not reflected by BMI alone. Lean NAFLD is also often underdiagnosed or diagnosed at more advanced stages, potentially leading to delayed intervention and poorer outcomes. Additionally, lean individuals may experience under-recognition of cardiovascular or metabolic risk, contributing further to increased mortality. These diverse pathways suggest that BMI alone does not capture the full spectrum of risk in NAFLD and underscore the need for individualized risk stratification. Interestingly, the lack of increased cardiovascular or cancer mortality further underscores the idea that, in lean NAFLD, liver-specific mechanisms, rather than systemic metabolic burden, may be the primary drivers of poor prognosis.

### Implications of the findings for clinical practice:

Our findings highlight the need to reconsider the clinical approach to lean NAFLD. Despite the absence of obesity, lean individuals with NAFLD are at higher risk of all-cause and liver-related mortality, indicating that this phenotype is not benign. Clinicians should maintain a high index of suspicion and routinely assess liver disease severity in lean patients, including the use of non-invasive fibrosis markers or imaging when appropriate. Risk stratification tools should move beyond BMI to incorporate measures of visceral adiposity, sarcopenia, genetic susceptibility and liver-specific biomarkers. Although lean patients may have fewer metabolic abnormalities, their comparable risk of cardiovascular and cancer-related mortality suggests the need for comprehensive assessment and preventive care in these domains. Overall, lean NAFLD should be recognized as a distinct clinical entity requiring early diagnosis, individualized monitoring, and tailored interventions to improve outcomes and reduce mortality.

### Limitations:

Firstly, observational nature of the included studies restricted our ability to establish causality. Secondly, substantial heterogeneity was noted, leading to the utilization of a random-effects model for analysis. Despite conducting subgroup analyses based on various factors, such as study design, sample size, follow-up duration, study setting, and methods for NAFLD diagnosis, certain sources of heterogeneity, including metabolic profiles, NAFLD severity, and genotypes influencing liver metabolism, could not be thoroughly evaluated. Thirdly, although the inclusion criteria focused on studies reporting adjusted effect sizes, there is still a risk of residual confounding by unmeasured factors. Fourthly, there was ambiguity in categorizing subjects as “lean” or “non-lean” within the studies, including the inclusion of normal and underweight individuals in the “lean” category and overweight and obese subjects in the non-lean category.

## CONCLUSION

This review highlights the increased risk of all-cause mortality and liver disease-related mortality in lean patients with NAFLD. Our findings emphasize the need for clinicians to reconsider assumptions about protective effects in lean individuals and extend monitoring and interventions to both lean and non-lean populations. Future research should focus on the underlying mechanisms contributing to increased mortality risk in lean NAFLD patients, exploring genetic factors, metabolic profiles, and the impact of visceral adipose tissue.

### Future directions:

Future research efforts should prioritize understanding the underlying mechanisms contributing to the increased mortality risk observed in lean individuals with NAFLD. Longitudinal studies with extended follow-up periods are essential to comprehensively assess the dynamic nature of mortality risks over time. Additionally, prospective studies exploring the effectiveness of tailored interventions and treatment strategies for lean NAFLD patients are needed to inform evidence-based clinical guidelines and improve patient outcomes.

### Authors’ contributions:

**PM, ZS and JJ:** Literature search, study design and manuscript writing.

**ZS and JJ:** Data collection, data analysis and interpretation. Critical review.

**PM:** Literature search, manuscript revision and validation. Critical analysis.

All authors have read, approved the final manuscript. They are also accountable for integrity of the study.
